# The Rejuvenation Effect of Bio-Oils on Long-Term Aged Asphalt

**DOI:** 10.3390/ma17133316

**Published:** 2024-07-04

**Authors:** Jintao Wang, Shi Xu, Sifan Zhu, Qin Tian, Xinkui Yang, Georgios Pipintakos, Shisong Ren, Shaopeng Wu

**Affiliations:** 1 School of Civil Engineering and Architecture, Wuhan University of Technology, Wuhan 430070, China; 2 Faculty of Civil Engineering and Geosciences, Delft University of Technology, 2628 CN Delft, The Netherlands; 3 Hubei Key Laboratory of Roadway Bridge and Structure Engineering, Wuhan University of Technology, Wuhan 430070, China; 4 State Key Laboratory of Silicate Materials for Architectures, Wuhan University of Technology, Wuhan 430070, China; 5 SuPAR Research Group, University of Antwerp, 2020 Antwerp, Belgium

**Keywords:** asphalt, aging, rejuvenating, sunflower oil, soybean oil, palm oil, physical and rheological properties, chemical composition

## Abstract

Generally, rejuvenators are used to supply missing components of aged asphalt, reverse the aging process, and are widely used in asphalt maintenance and recycling. However, compared with traditional rejuvenators, bio-oil rejuvenators are environmentally friendly, economical and efficient. This study looks into the effect of the three different bio-oils, namely sunflower oil, soybean oil, and palm oil, on the physical properties, rheological properties and chemical components of aged asphalt at different dosages. The asphalt physical properties and Dynamic Shear Rheological (DSR) test results show that with the increase in bio-oil, the physical properties and rheological properties of rejuvenated asphalt are close to those of virgin asphalt, but the high-temperature rutting resistance needs to be further improved. The results of Fourier Transform Infrared Spectroscopy (FTIR) show that the carbonyl and sulfoxide indices of rejuvenated asphalt are much lower than those of aged asphalt. Moreover, the rejuvenation efficiency of aged asphalt mixed with sunflower oil is better than that with soybean oil and palm oil at the same dosage.

## 1. Introduction

Asphalt pavement is becoming increasingly popular for its advantages of convenient construction, waterproofing, skid resistance and comfort driving experience [1]. However, aging takes place during the mixing, production and long-term service of asphalt pavement, causing the asphalt to become brittle and therefore inducing undesirable phenomena such as potholes and cracks, ultimately resulting in the end of its service life [2,3,4]. Zhang et al. [5] studied the diffusion influence mechanism of virgin and aged asphalt binder systems using molecular dynamics, and revealed the aging mechanism of asphalt. Asphalt rejuvenators can be used to reverse this aging process, which is a practice widely used in the maintenance and recycling of asphalt concrete [6]. In industry, rejuvenators are mainly produced from mineral or aromatic oils. Mineral oils are prone to volatilization at higher recovery temperatures and their poor stability will adversely affect the efficiency of rejuvenators. Aromatic oil has a relatively lower rejuvenation efficiency, and it also contains higher carcinogenic and polluting polycyclic aromatic hydrocarbons, which can be harmful to human health and natural surroundings [7,8,9]. As a result, it is essential to explore an alternative, more efficient and environmentally friendly asphalt rejuvenator for the road industry [10,11].

Bio-oil is a renewable material that can be derived through pyrolysis from crops, cotton, corn, waste wood and others [12,13]. It boasts a broad range of raw materials, low production costs, environmental friendliness, and renewability [14], and its composition is close to the light components of asphalt. Adding bio-oil to the aged asphalt can supplement the light components lost during the aging process, achieve the coordination of components and the recovery of various properties. [15,16]. Yan et al. [17] pointed out that the use of tung oil can greatly improve the flexibility and low-temperature crack resistance of aged asphalt, and appropriate contents of tung oil can ensure the high-temperature performance and lower temperature sensitivity of rejuvenated asphalt. The bio-oil obtained from Jatropha curcas is feasible as an asphalt rejuvenator in terms of environmental protection and rejuvenation efficiency, while the rejuvenated asphalts become very stable in storage [18]. Samieadel et al. [19] prepared biological rejuvenators by mixing high-protein algae with high-fat animal feces, and discovered that the high-protein algae contributed to chemical balance, while the high-fat animal feces were conductive to depolymerizing asphaltenes. Gong et al. [20] found that bio-oil derived from waste wood and pig feces restored short-term aged PEN50 to the level of virgin asphalt. Bio-oils obtained from waste wood resources can significantly improve asphalt fatigue performance and can be a suitable modifier for petroleum asphalt binders in the pavement industry [21]. Moisture weakens the bonding force between the aggregate and the asphalt, which causes the asphalt to peel off the surface of the aggregate [22]. However, it was found that the use of bio-oils as a partial replacement for asphalt can improve adhesion with aggregates and increase moisture damage resistance [23]. Moreover, the use of biobinders, which is greatly effective in hot recycling, can improve the crack resistance of the mixture [24]. The above research results not only confirm that the bio-oil rejuvenator has a good rejuvenation effect but also provide a beneficial exploration of new ways to obtain the bio-oil rejuvenator. 

Bio-oils being used so far have achieved promising rejuvenation effects on aged asphalt, but additional procedures such as extraction or necessary pretreatment hinder the wide spread of these biomaterials. The bio-oil represented by sunflower oil, palm oil and soybean oil has been considered a green and sustainable rejuvenator product for ameliorating the performance of aged asphalts due to their wide range of raw material sources and acceptable rejuvenation effect [25,26,27]. Shirzad et al. [28] used sunflower oil as an asphalt rejuvenator to reverse the aging of the asphalt binder, and the tests showed a positive effect on both high-temperature and low-temperature grades of the asphalt. Rafiq et al. [29] found that the addition of different proportions of hairy palm oil to the aged RAP binder was able to soften the RAP binder as well as lower the viscosity. Elkashef et al. [30] mixed 6% soybean oil with reclaimed asphalt pavement (RAP) to study the performance of rejuvenated RAP and found that rejuvenated RAP has lower stiffness and greatly higher fatigue resistance. Although the current research believes that the above three bio-oil rejuvenators have a good rejuvenation effect, there is a lack of comparative research on the rejuvenation effect of these three bio-oil rejuvenators in aged asphalt.

In this study, three types of rejuvenators, namely sunflower oil, soybean oil and palm oil, were investigated. Firstly, a rolling thin film oven (RTFO) and a pressure aging vessel (PAV) were used to prepare the laboratory-aged asphalt [31,32], and then the aged asphalt samples were rejuvenated with the three bio-oils at different dosages. Finally, the rejuvenation effect and characteristics of these three bio-oils on the physical properties, rheological properties and chemical components of aged asphalt were investigated by asphalt physical property tests, Dynamic Shear Rheology (DSR) test and Fourier Transform Infrared Spectroscopy (FTIR) analysis.

## 2. Materials and Methods

### 2.1. Asphalts and Rejuvenators

The asphalt used in this study is virgin asphalt PEN70 (referred to as 70#), which is widely used in pavement engineering, and the measured physical properties are shown in Table 1.

**Figure 1:**
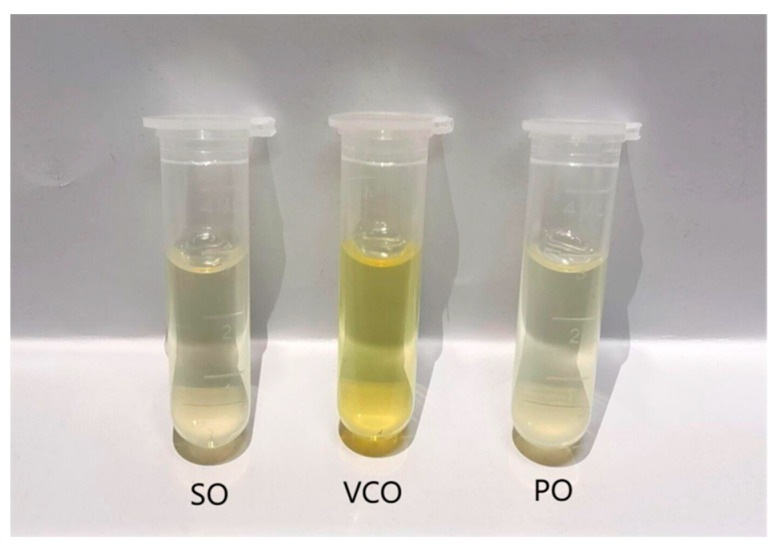
The appearance of the three bio-oils.

**Figure 2:**
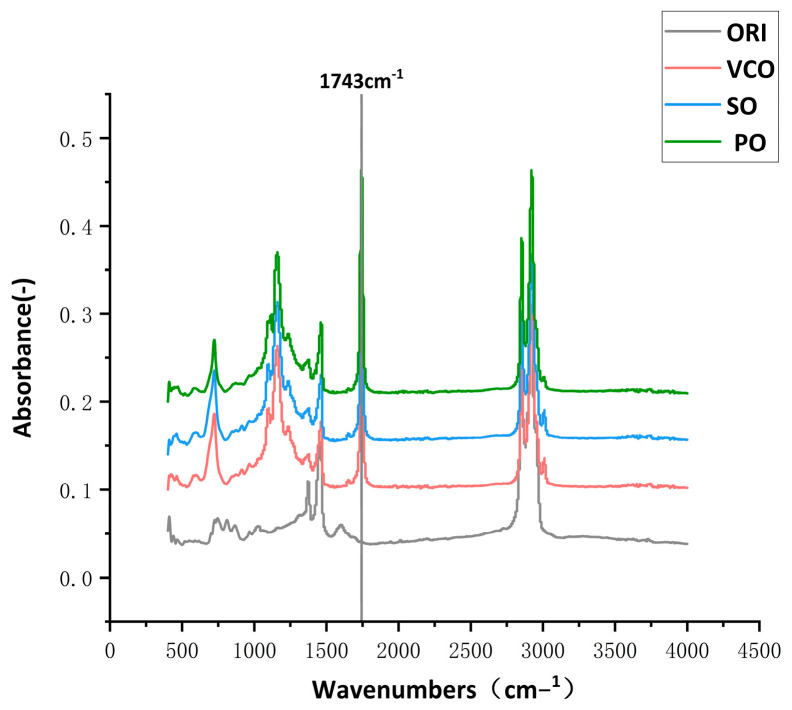
FTIR spectra of the three bio-oils and virgin asphalt.

Figure 1 shows the appearance of the three different bio-oil rejuvenators. Sunflower oil is presented in a transparent yellow liquid, while soybean oil and palm oil are shown as light-yellow liquids. The FTIR results of the three bio-oil rejuvenators and the virgin asphalt are shown in Figure 2. Comparing the spectra of the three bio-oils and the virgin asphalt, it was found that there is an evident difference between them. As can be seen from Figure 2, the virgin asphalt shows no apparent peak at approximately 1743 ${\mathrm{cm}}^{-1}$, while all three bio-oils have distinct characteristic absorption peaks, which are typically associated with the stretching vibration of the carbonyl groups (C=O) [17], indicating that the three bio-oils have similar functional groups. The virgin asphalt, sunflower, soya and palm oils are referred to in the text as ORI, VCO, SO and PO.

### 2.2. Rejuvenator Evaluations

#### 2.2.1. Asphalt Aging and Rejuvenation Tests

Following ASTM-D2872 [31], a rolling thin film oven (RTFO) was used to simulate the aging of asphalt during its production process, which refers to the short-term aging process. In the short-term aged test, cylindrical glass bottles containing virgin asphalt were stored in a rotating film oven for 85 min, and the oven temperature was maintained at 163 °C. In accordance with ASTM-D6521 [32], a pressure aging vessel (PAV) was used to simulate the aging of asphalt after 5–10 years of in-service, which refers to the long-term aging process. In the long-term aged test, aged asphalt samples from the RTFO test were placed in a PAV and then heated at a pressure of 2.1 MPa and a temperature of 100 °C for 20 h. Short-term aged asphalt and long-term aged asphalt are referred to as STA and LTA in the figure below.

**Figure 3:**
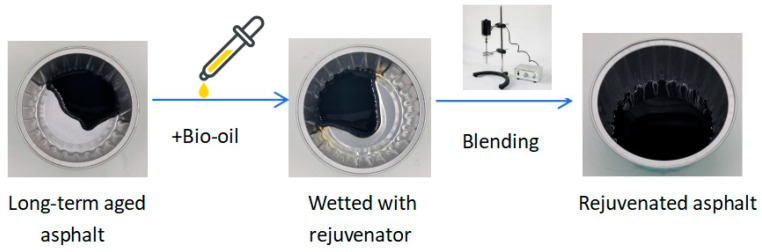
The procedure of aged asphalt rejuvenation.

Rejuvenated asphalt was formed by mixing asphalt after long-term aging with bio-oil. Figure 3 illustrates the rejuvenation process of aged asphalt. For this purpose, the long-term aged asphalt samples were loaded into aluminum cups and all bio-oil rejuvenators were dripped on the asphalt sample at dosages of 2%, 4%, 6% and 8%. Then, samples were mixed well with a glass rod and continuously sheared with a mixer at a shear rate of 300 rpm under 140 °C for 20 min, and finally, the rejuvenated asphalt was obtained.

#### 2.2.2. Physical Property Tests

The physical properties of asphalt significantly impact the performance of asphalt pavement. In this study, the optimum bio-oil dosages for aged asphalt rejuvenation are mainly determined by the asphalt’s physical properties, including penetration level and softening point, which are tested following ASTM-D5 [33] and ASTM-D36 [34], respectively.

#### 2.2.3. Rheological Property Tests

A Dynamic Shear Rheometer (DSR) MCR101 (Anton Par, Canberra, Australia) was used to study the rheological properties of the asphalt samples. The complex shear modulus (G*), phase angle (δ) and rutting parameter (G*/sinδ) of the asphalt samples at different temperatures were obtained through the temperature scanning program to study the changes in viscoelastic properties and high-temperature performance of the asphalt during the aging and rejuvenation process [35]. The temperature scanning program was carried out at temperatures increased from 30 °C to 80 °C, using a frequency of 10 Hz. The test was performed with 25-mm parallel plates, and the asphalt samples had a thickness of 1 mm, which was conducted in strain control mode with a maximum shear strain value of 1.2%.

#### 2.2.4. Chemical Property Tests

Asphalt aging causes a change in the chemical structure, which can be found in an increase in carbonyl groups (C=O) and sulfenyl groups (S=O); therefore, the characteristic functional groups such as carbonyl groups and sulfenyl groups are selected in the Fourier Transform Infrared Spectroscopy (FTIR) scanning results [7], the peak areas are calculated, and the carbonyl index (*Ic*) and sulfoxide index (*Is*) are obtained with the aid of the OMNIC Infrared Data Processing Software (OMNIC 9.2.86) to reflect the extent of the influence of the aging and rejuvenation conditions on the internal components and structure of asphalt [36,37]. The test was performed in transmission mode, and each asphalt sample was scanned 32 times with a resolution of 4 ${\mathrm{cm}}^{-1}$.

(1) $$I_{C}=\frac{A_{1696}}{\sum A}$$

(2) $$I_{S}=\frac{A_{1030}}{\sum A}$$

(3) $$\sum A=A_{725}+A_{746}+A_{810}+A_{865}+A_{1030}+A_{1373}+A_{1456}+A_{1601}+A_{1696}+A_{2853}+A_{2921}$$

where: $A_{i}:$ is the peak area at the corresponding wave number of the characteristic functional group *i*.

**Figure 4:**
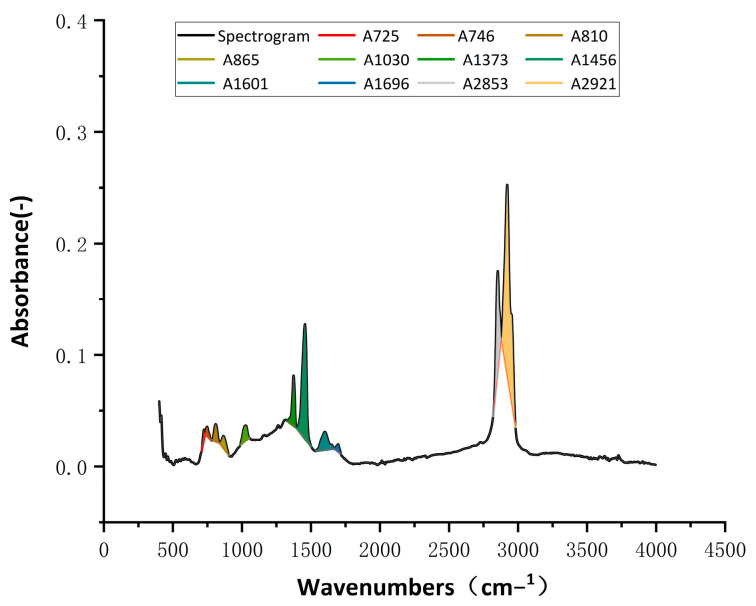
The reference areas of FTIR spectra.

Learning from the experience of Lamontagne et al. [38], the reference area (∑A) calculated in this study is obtained on the basis of the asphalt characteristics and shown in Figure 4 with different colors. Each color area represents the peak area ($A_{i}$) of a certain characteristic functional group of the asphalt.

## 3. Results and Discussion

### 3.1. Physical Properties

**Figure 5:**
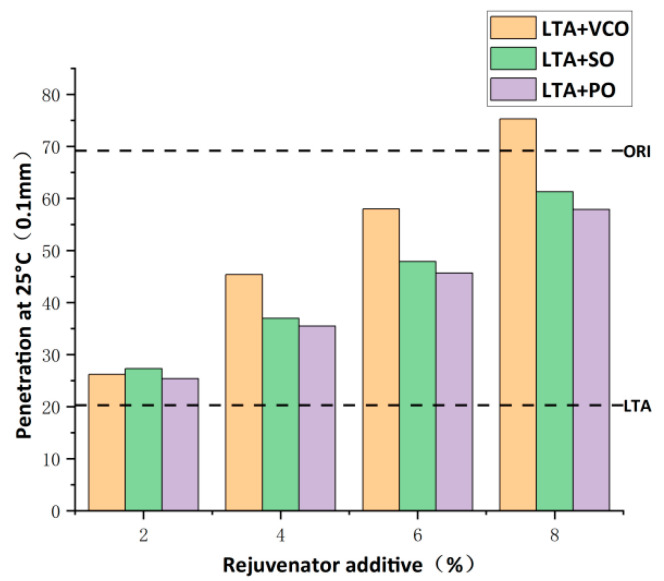
Penetration of rejuvenated asphalt with the three bio-oils at different dosages.

Figure 5 shows the effect of the three bio-oils at four different dosages on the penetration of aged asphalts at 25 °C. VCO rejuvenated asphalt, SO rejuvenated asphalt, and PO rejuvenated asphalt are referred to as LTA+VCO, LTA+SO, LTA+PO in the figure below. It is clear from Figure 5 that the penetration of the PEN70 virgin asphalt decreases sharply from 69.2 (0.1 mm) to 20.3 (0.1 mm). The penetration of the aged asphalt is significantly restored by the addition of these three bio-oils, and the penetration value grows steadily with the addition of the three bio-oils. In addition, the softening effect of the three bio-oils in restoring the penetration of aged asphalt at the same dosage is decreased by VCO, SO and PO. The rejuvenator dosage for SO and PO is found at 8% when the rejuvenated asphalt has a penetration close to the virgin asphalt, while the penetration value of VCO is even higher than the virgin asphalt. Therefore, the optimum dosage of VCO to restore the penetration of aged asphalt is no more than 8%, and those of SO and PO are not less than 8%. 

**Figure 6:**
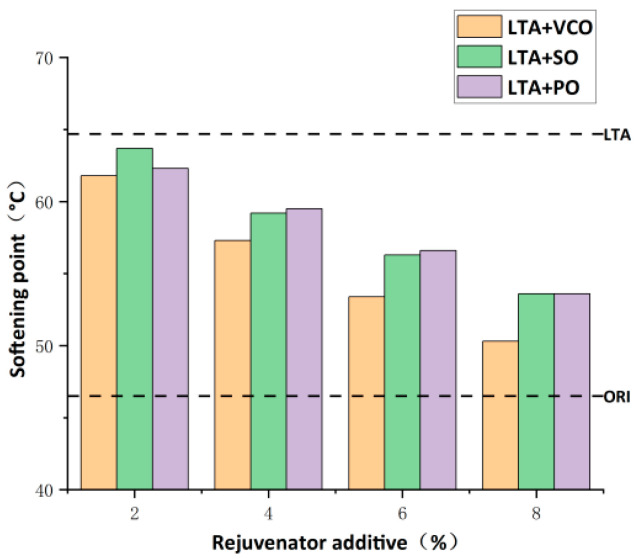
Softening point of rejuvenated asphalt with the three bio-oils at different dosages.

The softening point reflects the consistency at high temperatures and the temperature stability of asphalt. The temperature sensitivity decreases with an increase in the softening point [39]. Figure 6 shows the effect of the three bio-oils at four different dosages on the softening point of aged asphalts. The softening point of PEN70 virgin asphalt increases from 46.5 °C to 64.7 °C after aging, which shows that the asphalt becomes harder and has better high-temperature stability during the aging process. The addition of all three bio-oils reduces the softening point of aged asphalt, and the softening point value of aged asphalt gradually decreases with the increase in the three bio-oils, indicating that the bio-oil rejuvenators soften the aged asphalt as in the penetration test, but the resistance to high-temperature deformation is reduced, so the dosage of the bio-oil rejuvenator should be controlled within a reasonable range. VCO has the highest rejuvenation efficiency under the same dosage, and SO and PO have comparable rejuvenation efficiencies. When the dosage of the three bio-oils reached 8%, the softening point of the rejuvenated asphalt was closest to that of the virgin asphalt, so the optimum dosage of VCO, SO and PO to restore the softening point of aged asphalt was not less than 8%.

On the basis of the above test results, the optimum dosage of VCO is 8%, and the optimum dosages of SO and PO are both not less than 8%. For SO and PO, when the dosage reaches 8%, the penetration and the softening point of the rejuvenated asphalt are close to those of the virgin asphalt, which means that the two bio-oils already have a good rejuvenation efficiency, and in order to reduce the cost, the optimum dosages of SO and PO are also 8%. In summary, the optimum dosages of all three bio-oils are determined to be 8%.

### 3.2. Rheological Properties

#### 3.2.1. Effect of Aging on the Rheological Properties

**Figure 7:**
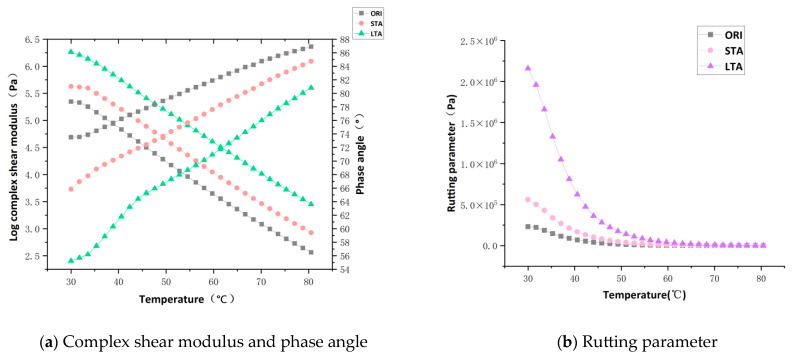
Complex shear modulus, phase angle and rutting parameters for virgin asphalt and aged asphalt.

Figure 7 shows the complex shear modulus, the phase angle and the rutting parameter measured at temperatures increased from 30 °C to 80 °C for the asphalt with different degrees of aging. The complex shear modulus reflects the ability of asphalt to resist shear strain [40], the phase angle characterizes the delay of the mechanical response between the viscous and elastic parts of the asphalt [39], and the rutting parameter indicates the capability of asphalt pavement to resist permanent deformation [20]. As can be seen from Figure 7, with the extension of the aging time of asphalt, the value of complex shear modulus and rutting parameter increases, and the value of the phase angle gradually decreases, which is due to the aging that changes the viscoelastic properties of asphalt, so that the elastic component of asphalt increases and the viscous component decreases, and the rutting resistance is improved [41].

#### 3.2.2. Effect of Rejuvenator Dosages on Rheological Properties

**Figure 8:**
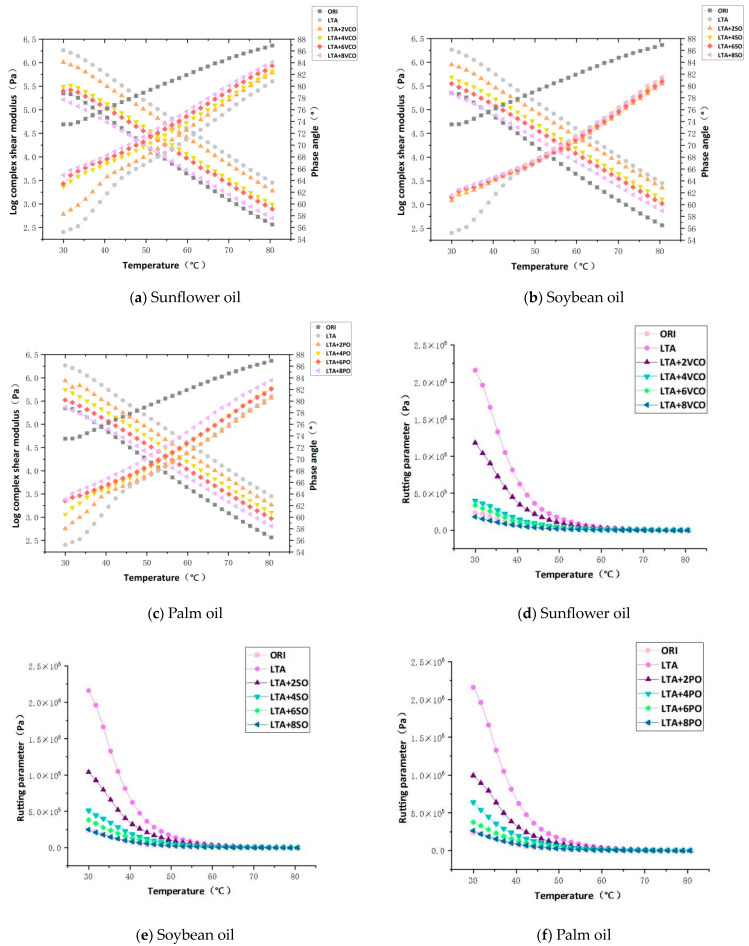
Complex shear modulus, phase angle and rutting parameter for rejuvenated asphalt.

As can be seen from Figure 8, the complex shear modulus, the phase angle and the rutting parameter of the long-term aged asphalt are restored to a certain extent when three bio-oils are added to the aged asphalt, indicating that aged asphalt gets a certain degree of softening and the viscous components are increased, but the deformation resistance is weakened. The complex shear modulus of aged asphalt declines as the content of the three bio-oils increases. When the dosage of the three bio-oils is 8%, the value of rejuvenated asphalt is close to that of virgin asphalt, which is in line with the conclusion obtained from the asphalt penetration and softening point tests. With the increase in VCO and PO, the phase angle of aged asphalt increases obviously, and the rejuvenation efficiency is better. However, the phase angle of aged asphalt is basically unchanged as SO increases, indicating that SO has little effect on the phase angle of aged asphalt.

As can be seen from Figure 8d–f, the rutting parameter value of rejuvenated asphalt gradually decreases with the increase in the three bio-oils. This is due to the change in the proportion of asphalt components after the addition of bio-oil, and the lost light component is replenished, resulting in better fluidity and deformation capacity of asphalt at high temperatures, which leads to a decline in rutting parameters [7], so the dosage of the three bio-oils needs to be controlled within a reasonable range. In addition, the rutting parameter decreases faster in the low-temperature region, while the decline trend tends to be gentle at higher temperatures. The rutting parameter of rejuvenated asphalt at other dosages is higher than that of virgin asphalt except for the dosage of 8%, which indicates that the rejuvenated asphalt with the appropriate content of bio-oils still has better high-temperature stability; therefore, from the perspective of the rutting parameter, the optimum dosage of the three bio-oils should not exceed 8%.

#### 3.2.3. Effect of Types of Rejuvenators on Rheological Properties

Table 2, Table 3 and Table 4 shows the complex shear modulus, the phase angle and the rutting parameter of rejuvenated asphalt with the integer temperature point at the optimum dosage of the three bio-oils. It can be seen that the efficiency of the three bio-oils in restoring the complex shear modulus of aged asphalt in the low-temperature region is decreased as VCO, SO and PO, and the complex shear modulus of rejuvenated asphalt is lower than that of the virgin asphalt, while the rejuvenation efficiency of the complex shear modulus of aged asphalt is decreased as VCO, PO and SO in the high-temperature region. At any temperature, the efficiency of the three bio-oils to restore the phase angle of aged asphalt is decreased by VCO, PO and SO.

It can be clearly seen that the rutting parameter of the rejuvenated asphalt after the addition of SO and PO is close to that of the virgin asphalt. In the low-temperature region, the weakening effect of SO on the rutting resistance of aged asphalt is greater than that of PO, while the opposite is true in the high-temperature region. The rutting parameter of VCO-rejuvenated asphalt is always lower than that of SO-rejuvenated asphalt and PO-rejuvenated asphalt, which means the rejuvenation efficiency of VCO is superior to that of SO and PO in terms of rheological properties. Compared with SO and PO, VCO has the most obvious weakening of rutting resistance in aged asphalt.

### 3.3. Chemical Properties

#### 3.3.1. Effect of Aging on the Chemical Composition

**Figure 9:**
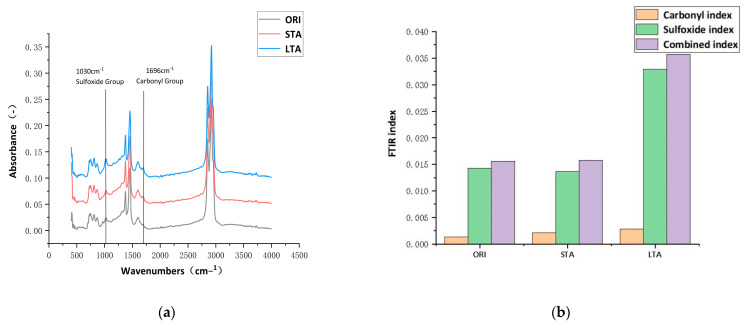
FTIR spectra and FTIR index of aged asphalt and virgin asphalt.

Figure 9a shows the FTIR spectra of aged asphalt and virgin asphalt, and Figure 9b is the FTIR index of aged asphalt and virgin asphalt. As can be seen from Figure 9, the changes in the spectra of asphalt after aging mainly occur in the peak area of some characteristic functional groups. Among them, the area of the sulfoxide group characteristic peak at 1030 ${\mathrm{cm}}^{-1}$ and the carbonyl group characteristic peak at 1696 ${\mathrm{cm}}^{-1}$ increase, indicating that the chemical structure of the asphalt changes during the aging process, and the C-C and C-H bonds in asphalt are broken down to create free radicals, which triggers a series of chemical reactions such as condensation and dehydrogenation of asphalt molecules. These chemical reactions result in the formation of more carbonyl and sulfoxide groups in the asphalt molecule [42], ultimately showing an increase in two FTIR indices after aging.

#### 3.3.2. Effect of Dosages and Types of Rejuvenators on the Chemical Composition

**Figure 10:**
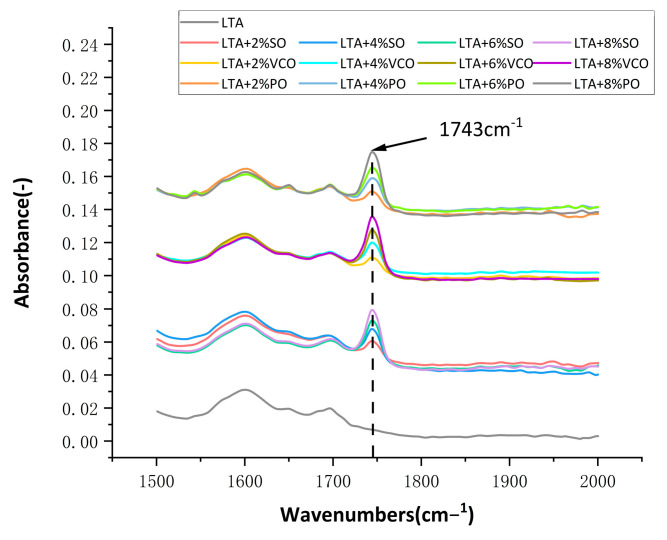
FTIR spectra of rejuvenated asphalt and long-term aged asphalt.

Figure 10 shows the infrared spectra of rejuvenated asphalt and long-term-aged asphalt. It can be seen that the rejuvenated asphalt only has a new characteristic peak at 1743 ${\mathrm{cm}}^{-1}$, which is the characteristic peak of the three bio-oils, and the area of the peak increases with the increase in bio-oils.

**Figure 11:**
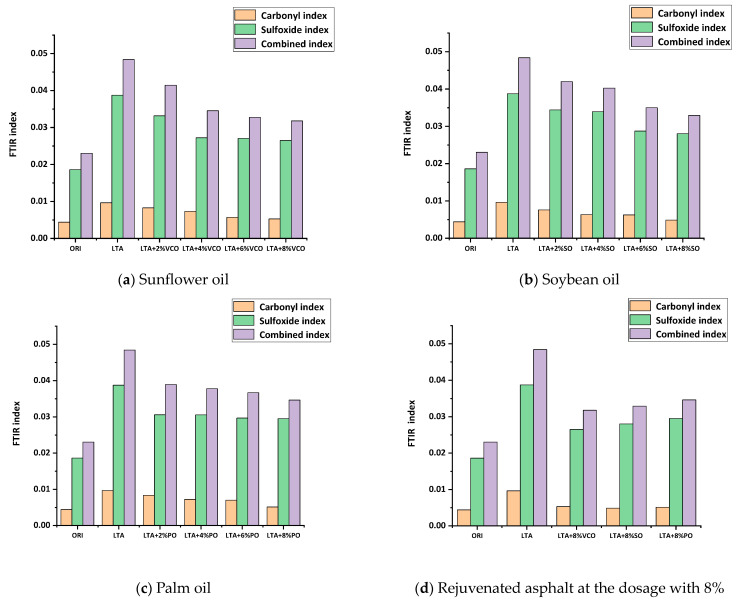
FTIR index of rejuvenated asphalts.

Figure 11 shows the FTIR indices of aged asphalt blended with sunflower oil, soybean oil and palm oil, respectively. Figure 11a–c indicate that the FTIR indices of rejuvenated asphalt gradually decrease with the increase in bio-oils, indicating that the three kinds of bio-oils can improve the aging of aged asphalt, which could be due to the combination of physical effects (dissolution and diffusion) and chemical effects (scavenging free radicals and inhibiting asphaltene aggregation) [43]. When the dosage is 8%, the FTIR indices of the three kinds of rejuvenated asphalt are close to those of the virgin asphalt.

Figure 11d shows the comparison of rejuvenation effects for all three types of bio-oils at a dosage of 8%. It shows that when the dosage is 8%, the FTIR indices of the three kinds of rejuvenated asphalt, from smallest to largest, are VCO rejuvenated asphalt, SO rejuvenated asphalt and PO rejuvenated asphalt, implying that the efficiency of the three bio-oils in rejuvenating the chemical composition of the aged asphalt decreases sequentially from VCO, SO and PO.

## 4. Conclusions

The above results show that VCO, SO and PO all have a great effect on the long-term-aged asphalt, and several conclusions can be drawn:

1. The aging of PEN70 virgin asphalt decreases the penetration and increases the softening point, while the viscous component decreases and the elastic component increases, leading to a decrease in the phase angle and an increase in the complex shear modulus, and the rutting resistance becomes better. The presence of oxidized molecules in asphalt leads to an increase in carbonyl (C=O) and sulphinyl (S=O) groups.
2. The results of the asphalt physical properties test show that the addition of the three bio-oil rejuvenators can soften the long-term aged asphalt and restore the penetration and softening point of aged asphalt, and the rejuvenation efficiency of the three bio-oils on the physical properties of aged asphalt is decreased as VCO, SO and PO. When the content reaches 8%, the physical properties of the aged asphalt can be roughly rejuvenated to a level close to the virgin asphalt. Therefore, considering both economic benefits and rejuvenation efficiency, the optimal dosages of the three bio-oils are determined to be 8%.
3. DSR test results show that with the increase in the three bio-oils, the complex shear modulus and rutting parameter of the aged asphalt decrease, and the phase angle increases, indicating that the three bio-oils rejuvenate the rheological properties of the aged asphalt to a certain degree, but it is not conducive to the high-temperature stability of the asphalt.
4. Comparing the rejuvenation efficiency of the three oils on the complex shear modulus of aged asphalt, in the low-temperature region it is decreased as VCO, SO and PO, while the rejuvenation efficiency of the complex shear modulus of aged asphalt is decreased as VCO, PO and SO in the high-temperature region. There is always a sequential decrease in VCO, PO and SO when recovering the phase angle of aged asphalt. The rejuvenation efficiency of VCO on the antirutting performance of aged asphalt is more obvious than that of SO and PO. In the low-temperature zone, the rejuvenation of SO on the antirutting performance of aged asphalt is greater than that of PO, while the opposite is true in the high-temperature region.
5. The FTIR results show that the carbonyl index and sulfoxide index of aged asphalt decrease with an increase in the three bio-oils, indicating that the three bio-oils can improve the effect of aging on asphalt. In addition, the efficiency of the three bio-oils in rejuvenating the chemical composition at the optimum dosage decreases sequentially from VCO, SO and PO.
6. Based on the above conclusions, it is obvious that the rejuvenation efficiency of VCO on the physical properties, rheological properties and chemical components of aged asphalt is better than that of SO and PO, so VCO is more suitable to be a bio-oil rejuvenator.
7. Future research should pay more attention to the antiaging performance of rejuvenated asphalt and the green, sustainable, intelligent and automated production of bio-oil rejuvenators, so as to provide a basis for the comprehensive evaluation of bio-oil rejuvenators.

## Figures and Tables

**Table 1:** Physical properties of PEN70 virgin asphalt.

Property	Unit	Specification	Measured	Standard
Penetration at 25 °C	0.1 mm	60–80	69.2	ASTM-D5 [33]
Softening point	°C	≥45	46.5	ASTM-D36 [34]

**Table 2:** Log of complex shear modulus of rejuvenated asphalt at the dosage of 8% (Pa).

Temperature (°C)	ORI	LTA	LTA + 8VCO	LTA + 8SO	LTA + 8PO
30	5.35	6.07	5.22	5.33	5.34
40	4.83	5.74	4.74	4.88	4.89
50	4.28	5.21	4.28	4.45	4.44
60	3.65	4.61	3.72	3.91	3.87
70	3.08	4.01	3.19	3.38	3.33
80	2.56	3.45	2.70	2.87	2.81

**Table 3:** The phase angle of rejuvenated asphalt at the dosage of 8% (°).

Temperature (°C)	ORI	LTA	LTA + 8VCO	LTA + 8SO	LTA + 8PO
30	73.52	54.73	64.89	61.55	63.05
40	76.23	61.80	68.28	64.72	66.75
50	78.88	66.60	71.38	67.32	69.91
60	81.90	70.94	75.71	71.44	74.62
70	84.77	76.01	80.11	76.43	79.44
80	86.91	80.82	84.10	81.47	83.60

**Table 4:** The rutting parameter of rejuvenated asphalt at the dosage of 8% (Pa).

Temperature (°C)	ORI	LTA	LTA + 8VCO	LTA + 8SO	LTA + 8PO
30	232,000	1,260,000	182,000	251,000	264,000
40	70,110	624,000	58,765	84,107	85,104
50	19,601	178,000	20,165	30,341	29,226
60	4505	43,113	5465	8576	7739
70	1216	10,633	1590	2495	2156
80	366	2861	497	745	643

## Data Availability

The raw/processed date required to reproduce these findings cannot be shared at this time as the date also forms part of an ongoing study.
